# Decomposing Indigenous life expectancy gap by risk factors: a life table analysis

**DOI:** 10.1186/1478-7954-11-1

**Published:** 2013-01-29

**Authors:** Yuejen Zhao, Jo Wright, Stephen Begg, Steven Guthridge

**Affiliations:** 1 Health Gains Planning Branch, Northern Territory Department of Health, Darwin Plaza, 1st Floor, Smith St Mall, Darwin, NT 0801, Australia; 2 Research and Economic Analysis Unit, Queensland Health, 147-163 Charlotte St, Brisbane, QLD, 4000, Australia

**Keywords:** Health status disparities, Risk factors, Life expectancy, Indigenous population, Socioeconomic factors

## Abstract

**Background:**

The estimated gap in life expectancy (LE) between Indigenous and non-Indigenous Australians was 12 years for men and 10 years for women, whereas the Northern Territory Indigenous LE gap was at least 50% greater than the national figures. This study aims to explain the Indigenous LE gap by common modifiable risk factors.

**Methods:**

This study covered the period from 1986 to 2005. Unit record death data from the Northern Territory were used to assess the differences in LE at birth between the Indigenous and non-Indigenous populations by socioeconomic disadvantage, smoking, alcohol abuse, obesity, pollution, and intimate partner violence. The population attributable fractions were applied to estimate the numbers of deaths associated with the selected risks. The standard life table and cause decomposition technique was used to examine the individual and joint effects on health inequality.

**Results:**

The findings from this study indicate that among the selected risk factors, socioeconomic disadvantage was the leading health risk and accounted for one-third to one-half of the Indigenous LE gap. A combination of all six selected risks explained over 60% of the Indigenous LE gap.

**Conclusions:**

Improving socioeconomic status, smoking cessation, and overweight reduction are critical to closing the Indigenous LE gap. This paper presents a useful way to explain the impact of risk factors of health inequalities, and suggests that reducing poverty should be placed squarely at the centre of the strategies to close the Indigenous LE gap.

## Background

Understanding the causes of the life expectancy (LE) gap between Indigenous and non-Indigenous Australians is of paramount importance to achieving the Australian governments’ commitment to close the Indigenous health gap [1]. Between 2005 and 2007, the estimated Indigenous LE gap in Australia as a whole was 12 years for men and 10 years for women, whereas the Northern Territory (NT) Indigenous LE gap was 15 to 21 years, much greater than the national figures [2,3]. In population, the NT is the smallest Australian jurisdiction (1% of the total) with a vast landmass (17%) and the highest proportion of Indigenous people (30%). Although there was clear improvement in LE for NT Indigenous females over recent decades, the improvement in LE among males was slow, and the changes in LE gap between Indigenous and non-Indigenous males stagnated [4-6]. Previous research indicates that cardiovascular disease, genitourinary disease, diabetes, and respiratory disease are the leading contributors to the excess Indigenous mortality [5]. Australian governments have stated their determination to close the Indigenous LE gap within a generation, and halve the education and employment gap within a decade [1]. Insight into the contributions of various risk factors to the health inequalities could help focus resources on how best to reduce these differences.

The period LE at birth summarises the mortality experience of a population by assuming the age-specific probabilities of death in a given time period are applicable to a birth cohort. During a lifetime, socioeconomic disadvantage may have profound effects on population health. International studies indicate that differentials in LE are largely driven by socioeconomic status, education, family income, employment, and occupation [7,8] and reductions in mortality occur within the context of sustained economic growth and improved living conditions [9]. The Indigenous LE gap in the NT loosely resembles the LE differences between poor and rich countries [10] but is occurring within a single jurisdiction of Australia. There is international evidence to suggest that the poor, less educated, unmarried, and unemployed tend to have a much shorter LE [11]. Smoking- and alcohol-related cancers, stroke, and traffic accidents can explain up to 40% of these differences [12]. Decreasing tobacco consumption by about two cigarettes per day or increasing fruit and vegetable consumption by 30% could potentially increase LE by one year [13], while reducing obesity and giving up smoking could improve LE by 4 to 6 and 6 to 8 years, respectively [14]. In America, family income accounts for 38% of the mortality differentials between blacks and whites, and a further 31% is explained by smoking, systolic blood pressure, cholesterol level, body-mass index, alcohol intake, and diabetes [7].

Previous studies have shown that approximately 80% of the Indigenous LE gap was attributable to chronic diseases during 1996 to 2000 [5], and about 70% resulted from mortality in those aged over 45 years during 1984 to 2004 [6]. However, until now most explanations have focused on underlying causes of death and demographic factors. A gap decomposition analysis by risk factors could contribute to our knowledge of what drives health inequalities, and inform the development of better government health policy [15]. In order to develop a better understanding of the links between health risks and Indigenous LE gap, we adopt the LE decomposition approach by quantifying the contribution of common modifiable health risks to differences in LE between Indigenous and non-Indigenous NT residents. We also examine the age pattern of survivorship to assist with more targeted prevention strategies.

## Methods

The death records of the NT residents from 1986 to 2005 were extracted from the Australian Bureau of Statistics (ABS) mortality dataset, which includes age, sex, Indigenous status, area, and underlying causes of death. It is generally accepted that the NT has the most accurate and complete Indigenous death data in Australia [4]. Population denominators by age group, sex, and area were also taken from ABS. Indigenous population size was derived using the ABS experimental estimates [16]. The International Statistical Classifications of Diseases and Related Health Problems (ICD), 9^th^ and 10^th^ revisions, were mapped to the standard burden of disease and injury categories [17]. The original ICD codes and forward mapping tables were checked for consistency between the two revisions. Areas were grouped into socioeconomic disadvantage quintile groups according to the index of relative socioeconomic advantage and disadvantage (IRSAD) [18].

For a specific underlying cause of death (disease) *j*, the number of deaths attributable to the risk *i* is estimated by multiplying the total number of deaths by the population attributable fraction (*PAF*) [19,20], which is derived using

$$\text{PA}F_{j}^{i}=\frac{\sum_{k}P_{k}^{i}\left(RR_{k}^{\mathrm{ij}}-1\right)}{1+\sum_{k}P_{k}^{i}\left(RR_{k}^{\mathrm{ij}}-1\right)}$$

where *P*_*k*_^*i*^ represents prevalence of risk *i*, *RR*_*k*_^*ij*^ relative risk of cause of death *j* attributable to risk *i*, and *k* different levels of exposure by age, sex, Indigenous status, and in some cases, socioeconomic groups. As an example, the *PAF* estimates for mouth and oropharynx cancer deaths caused by alcohol consumption are illustrated in Table 1. The *RR* parameter is assumed to be constant across Indigeneity and time period. Separate *PAF*s were calculated for socioeconomic disadvantage, smoking, alcohol, obesity, pollution, and intimate partner violence (Table 2).

**Table 1:** Example of calculating population attributable fraction, alcohol for mouth and oropharynx cancer

**Indigenous status**	**Sex**	**Age group**	**Prevalence**			PAF*
			**Low**	**Hazardous**	**Harmful**	
Indigenous	male	0-14	0.00	0.00	0.00	0.00
Indigenous	male	15-19	0.26	0.05	0.08	0.34
Indigenous	male	20-24	0.43	0.08	0.14	0.46
Indigenous	male	25-34	0.43	0.19	0.13	0.48
Indigenous	male	35-44	0.46	0.10	0.12	0.45
Indigenous	male	>=45	0.12	0.15	0.25	0.56
Indigenous	female	0-14	0.00	0.00	0.00	0.00
Indigenous	female	15-19	0.08	0.11	0.06	0.28
Indigenous	female	20-24	0.14	0.18	0.10	0.39
Indigenous	female	25-34	0.13	0.08	0.14	0.43
Indigenous	female	35-44	0.07	0.05	0.34	0.61
Indigenous	female	>=45	0.00	0.00	0.30	0.57
…	…	…	…	…	…	…

* Relative risks of mouth and oropharynx cancers for low, hazardous, and harmful drinkers are 1.45, 1.85 and 5.39 respectively [17].

**Table 2:** Selected health risk and the representative group

**Selected risk (Short Name)**	**Representative group of other risk factors**
Socioeconomic disadvantage (Disadvantage)	Poverty, poor education, poor nutrition, low birth weight, poor hygiene, nutrition deficiency
Smoking	Tobacco use
Alcohol	Substance abuse
Obesity	Overweight, physical inactivity
Pollution	Climate change, occupational exposures
Intimate partner violence (Assault)	Domestic violence, child sexual abuse, unsafe sex

These risks were selected on the basis of breadth of coverage and to avoid duplications. For instance, because overweight, obesity, and physical inactivity are related, only obesity was chosen to represent this group (see Table 2). The six selected risks were assessed individually and in combination. We analysed the gap by risk factors in order to ascertain the magnitude of the impact and evaluate potential benefit of the risk reduction. This study recognized that the risk factors have cumulative effects on LE.

The *P* and *RR* data were collated from the best available sources based on literature review, including the most recent national burden of disease and injury study, NT fact sheets, and an NT report on health inequality [2,17,21-23]. The impact of socioeconomic disadvantage was estimated through the following outcomes: infectious disease, cancer, nutritional conditions, circulatory, respiratory, digestive, and urinary diseases [21].

LE at birth and the differences in LE between Indigenous and non-Indigenous population were examined for the four five-year periods from 1986 to 2005. The LE and the 95% confidence interval were estimated using standard abridged period life tables [24]. Let *e*^0^(*a*, 1), *e*^0^(*a*, 2) and *e*^0^(*a*) denote the estimated LE at age *a* for the Indigenous, non-Indigenous, and total population. The decomposition of LE difference by causes was implemented by the discrete approximation of the Vaupel-Canudas method (Appendix B in [15]). The difference in estimated LE at birth between the Indigenous and non-Indigenous populations can be decomposed as *n* mutually exclusive risks (*i*=1,2…,*n*):

$$\sum_{i=1}^{n}\left[{\bar{\rho }}_{i}e_{i}^{+}+\mathrm{cov}\left(\rho_{i},e^{0}\right)\right]\;F_{i}$$

where ${\bar{\rho }}_{i}$ represents the weighted average mortality improvement from risk *i*, *e*_*i*_^+^ is the weighted average number of life years lost attributable to risk *i*, the weight *F*_*i*_ = ∫ _0_^*ω*^*f*_*i*_(*a*) *da* with *f*_*i*_(*a*) referring to the proportion of life table deaths from risk *i* at age *a*, which is *f*_*i*_(*a*) = *μ*_*i*_(*a*)*l*(*a*), and *ω* is the maximum age group in the life table. The notation *l*(*a*) is the life table survival probability and *μ*_*i*_(*a*) is the probability of death owing to risk *i*, estimated by

$$\mu_{i}\left(a\right)=\mu \left(a\right)\frac{D_{i}\left(a\right)}{D\left(a\right)},$$

where *μ*(*a*) is the total probability of death and *D*(*a*) the total number of deaths at age *a*, *D*_*i*_(*a*) = *D*(*a*)*PAF*^*i*^(*a*). Note *PAF*^*i*^(*a*) is disease specific (*j*) and subject to different exposure levels (*k*) at age *a*. The average years of life lost attributable to risk *i* is given by

$$e_{i}^{+}=\int_{0}^{\omega }e^{0}\left(a\right)f_{i}\left(a\right)\mathrm{da}/F_{i},$$

and the average mortality improvement from risk *i* is

$${\bar{\rho }}_{i}=\int_{0}^{\omega }\rho_{i}\left(a\right)f_{i}\left(a\right)\;\text{da}/F_{i}$$

with *ρ*_*i*_(*a*) being estimated by

$$-\ln\left[\frac{\mu_{i}\left(a,2\right)}{\mu_{i}\left(a,1\right)}\right].$$

This approximation is used for estimating mortality reduction at the midpoint of the two populations. The covariance between *ρ*_*i*_ and *e*^0^, cov(*ρ*_*i*_, *e*^0^), is given by

$$\int_{0}^{\omega }\left[\rho_{i}\left(a\right)-{\bar{\rho }}_{i}\right]\left[e^{0}\left(a\right)-e_{i}^{+}\right]f_{i}\left(a\right)\text{da}/F_{i}.$$

The detailed methodology can be found in [15]. Due to the short LE and small Indigenous population in the NT, *ω* is set at 75 years in this study. This method is compared with and validated by the conventional Arriaga method [25,26]. The risk factor decomposition follows intuitively from the methodology developed for causal decomposition of LE differences [15,26], by substituting deaths attributable to health risks for causes of death. This method is well suited to revealing the potential causes of the Indigenous health gap attributable to risk factors.

Our analysis proceeded in three steps:

1. We began by constructing life tables by sex and Indigenous status for the four study periods;

2. The LE gap between Indigenous and non-Indigenous population was decomposed by the mutually exclusive dichotomous variable for each risk factor (univariate model); and

3. The LE gap was then decomposed by a dummy variable: whether any of the selected risks existed, assuming multiple competing risks coexisted simultaneously and independently (multivariate model).

In step 2 (univariate model), each death was split into two parts (yes=attributable to the risk; no=not attributable to it) by the *PAF* according to the underlying cause of death (disease) and level of exposure to the risk (age group, sex, and area related socioeconomic status). We attributed underlying causes of death to each risk factor one at a time by applying the *PAF* to derive the number of deaths attributable to the risk. The univariate model represented the impact of a single risk. However, a simple addition of all univariate effects may overestimate the combined impact of all coexisting risks. In step 3, we use the total number of deaths attributable to the single risk obtained in step 2 to estimate the total *PAF*^*i*^ by age group, sex, year, and Indigenous status. The combined *PAF* was then

$$1-\prod_{i}\left(1-\text{PA}F^{i}\right)$$

where $\prod_{i}\left(1-\mathrm{PA}F^{i}\right)$ is the proportion of mortality that cannot be attributed to any of the risk factors studied [17]. To evaluate the impact of multiple risks, we assumed independence between coexisting risks. Interaction terms between risk factors were not included because our interest was in estimating the average effect of each risk factor. In the multivariate model, individual risks were considered independent increments to the risk of mortality in conjunction with other coexisting risks. Changes in the risk of death from any single risk were assumed not to influence the risk of death from other risks. The multivariate model represented the joint impact of all the selected health risks. The *PAF* has been assumed to be constant over time, therefore an increased contribution of deaths caused by the risk indicates an increase of the risk.

## Results

**Figure 1:**
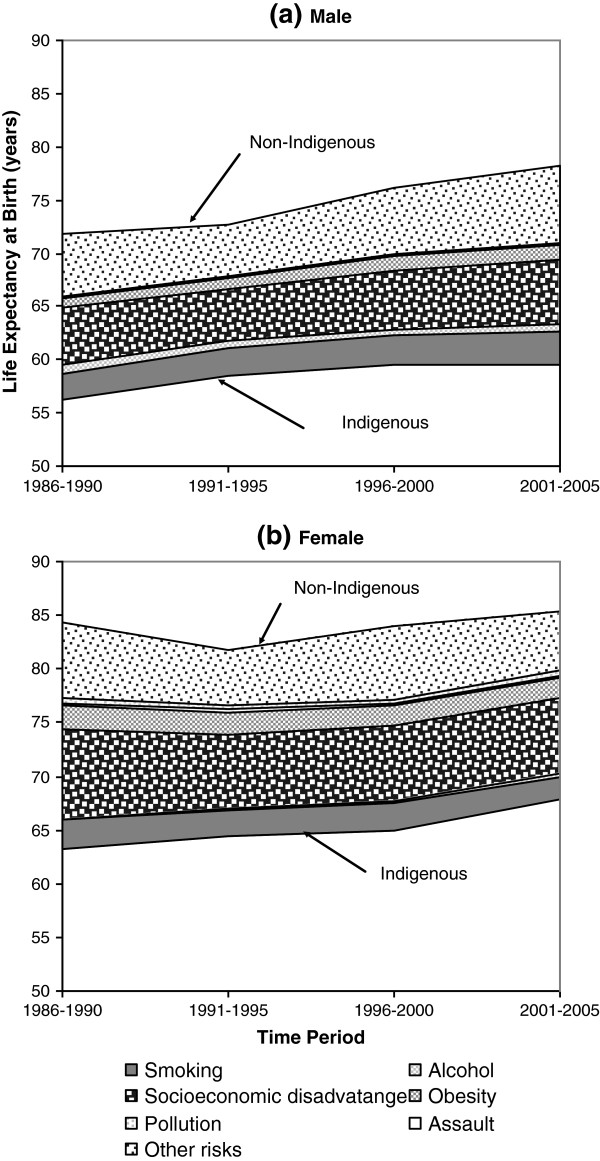
Indigenous life expectancy gap decomposed by six health risks for (a) male and (b) female, Northern Territory, Australia, 1986-2005.

Table 3 provides details of decomposed LE differences between NT Indigenous and non-Indigenous populations with 95% confidence intervals. During 1986 to 2005, Indigenous LEs were consistently lower than non-Indigenous expectancies. The gaps in LE at birth were large, being around 16 years for males and 19 years for females. Between 1996 and 2005, there was little improvement in the Indigenous male LE, which did not keep pace with its non-Indigenous counterpart (see Figure 1a). In contrast, the female LE gap narrowed markedly (see Figure 1b). Thus, it appears that the Indigenous LE gaps marginally deteriorated for males but improved for females over this period (P<0.05).

**Table 3:** Decomposed differences in life expectancy by health risks, Indigenous vs Non-Indigenous, Northern Territory, Australia, 1986–2005

	**LE**^**(a)**^**at birth**				**Contribution and CI**^**(c)**^			
**Health risk**	**1986-1990**	**1991-1995**	**1996-2000**	**2001-2005**	**1986-1990**	**1991-1995**	**1996-2000**	**2001-2005**
**Male**						%		
Smoking	3.25	3.41	3.75	4.01	21	24	23	21
Alcohol	1.08	0.83	0.80	1.02	7	6	5	5
Disadvantage	7.01	6.34	7.31	7.79	45	45	44	42
Obesity	1.35	1.46	1.86	1.77	9	10	11	9
Pollution	0.22	0.22	0.23	0.23	1	2	1	1
Assault	−0.02	−0.01	0.01	0.05	0	0	0	0
All-combined^(b)^	9.8	9.3	10.5	11.4	63	66	63	61
Unexplained	5.8	4.8	6.1	7.3	37	34	37	39
**LE**^**(a)**^**at birth**						**95% CI**^**(c)**^		
Indigenous	56.2	58.6	59.4	59.5	55.4-57.0	57.7-59.4	58.7-60.2	58.8-60.3
Non- Indigenous	71.8	72.7	76.1	78.3	71.2-72.3	72.2-73.1	75.6-76.6	77.8-78.7
Actual difference	15.6	14.1	16.6	18.8	14.8-16.3	13.4-14.8	16.0-17.3	18.1-19.4
**Female**						%		
Smoking	3.59	3.28	3.21	2.51	17	19	17	14
Alcohol	0.19	0.26	0.39	0.50	1	2	2	3
Disadvantage	11.17	9.31	9.11	8.97	53	54	48	51
Obesity	2.76	2.95	2.36	2.57	13	17	12	15
Pollution	0.33	0.32	0.27	0.20	2	2	1	1
Assault	0.63	0.52	0.37	0.56	3	3	2	3
All-combined^(b)^	14.1	12.2	12.1	11.9	66	70	64	68
Unexplained	7.1	5.2	6.9	5.6	34	30	36	32
**LE**^**(a)**^**at birth**						**95% CI**^**(c)**^		
Indigenous	63.2	64.4	65.0	67.9	62.4-64.0	63.6-65.3	64.2-65.7	67.1-68.7
Non- Indigenous	84.4	81.8	84.0	85.4	83.6-85.3	81.1-82.5	83.4-84.6	84.8-85.9
Actual difference	21.2	17.4	19.0	17.5	20.0-22.5	16.4-18.4	18.2-19.9	16.7-18.2

Notes: (a) LE=life expectancy, (b) All-combined model reflects the explanatory power of all risks combined and is not equivalent to the sum of the individual risks. Apportioning the combined overall risk back to each contributing risk factor would be highly sensitive to assumptions and is therefore not presented in this analysis, (c) CI=confidence interval. Data source: Northern Territory Department of Health, Australia.

Figure 1 shows LEs in Indigenous and non-Indigenous populations were improving differentially for males and females. LEs at birth in non-Indigenous males and Indigenous females improved much faster than those in Indigenous males and non-Indigenous females. The female gap was wider than the male gap by 5 years in 1986 to 1990. However, by 2001 to 2005 this trend was reversed and the female gap was one year narrower than the male gap. It is shown in Table 3 that the six selected risks jointly explained 60 to 70% of the Indigenous LE gap. In the univariate models, socioeconomic disadvantage was the single largest contributor, accounting for 42 to 54% of the Indigenous LE gap, followed by smoking (14 to 24%), obesity (9 to 17%), and alcohol (1 to 7%). Of the risk factors tested, the smallest contributors were assault and pollution, together accounting for 1 to 5% to the LE gap. The contributions of smoking and alcohol tended to be greater in males than in females, while the impacts of socioeconomic disadvantage and obesity were greater in females than in males. In terms of actual years, socioeconomic disadvantage contributed 9 to 11 years to the Indigenous LE gap for females, and 6 to 8 years for males. Smoking explained 3 to 4 years, obesity 1 to 3 years, and alcohol 0.2 to 1 year. Assault and pollution together contributed approximately one-fifth to one year. The contribution from assault was small but negative for Indigenous male LE gap for the period 1986 to 1995, indicating a competing or negative effect on the LE gap in males. Assault accounted for one-third to one-half of a year for the female LE gap. These results were almost identical to those of the Arriaga method. Approximately one-third of the Indigenous LE gap could not be explained by the multivariate model using the selected set of risk factors. This residual represents risk factors not included in our analysis, such as residence in a remote location, high incarceration rates, and a lack of health care access by the Indigenous population [27].

**Figure 2:**
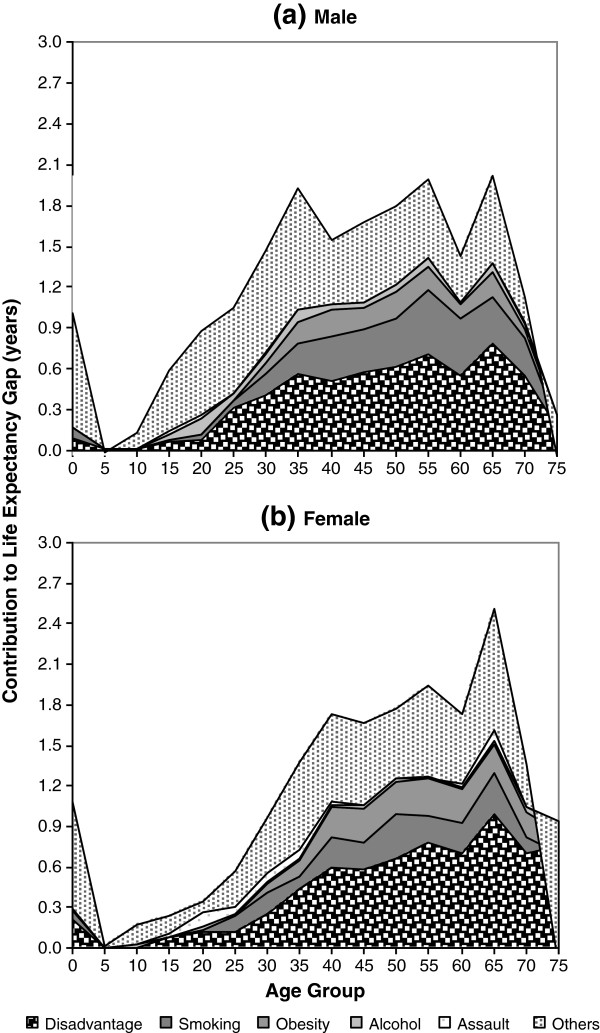
Contribution of risk factors to Indigenous life expectancy gap for (a) male and (b) female by age group, 2001-2005.

Figure 2 demonstrates the contributions of the risk factors to the Indigenous life expectancy gap by age groups between 2001 and 2005. Clearly, the majority (85%) of the health risks that contribute to the LE gap were concentrated among people older than 35 years. The age and sex patterns of socioeconomic disadvantage, smoking, and obesity appear to be consistent with the age and sex patterns of the total LE gap.

**Figure 3:**
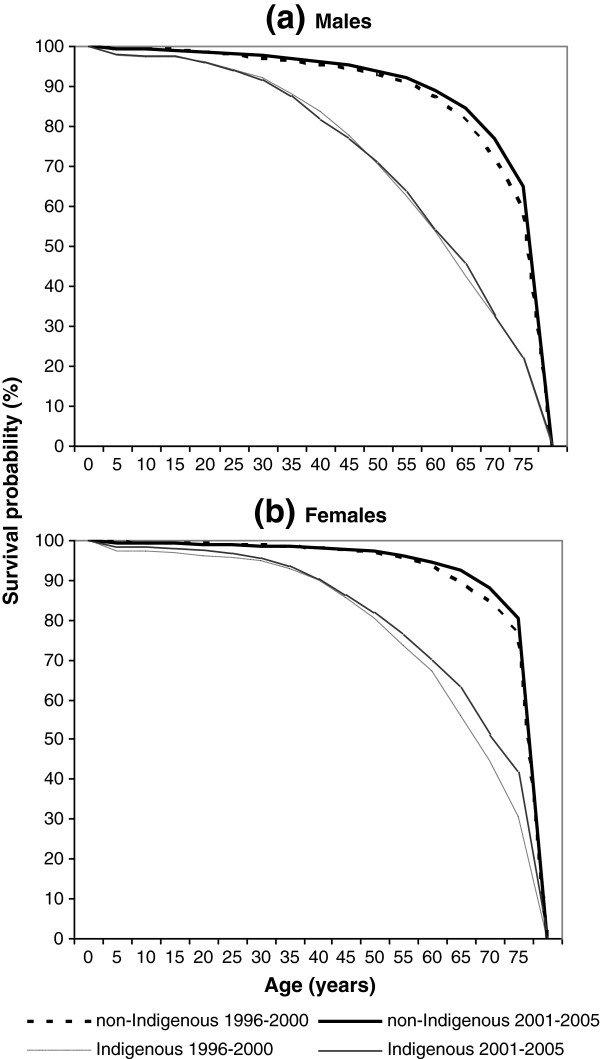
Survival curves by Indigenous status, (a) male and (b) female, Northern Territory, Australia, 1996–2000 vs 2001–2005.

Figure 3 demonstrates a shift of the survival curves to the upper-right corner (rectangularisation) between 1996 to 2000 and 2001 to 2005 for all groups except Indigenous males. There were encouraging improvements across all age groups for Indigenous females, with a substantial lift in survival in older women. The improvements are visible across most age groups for non-Indigenous males, but only limited improvement was observed for non-Indigenous females and this was restricted to mortality in the elderly. By examining the Indigenous male survival curves in detail (see the thin lines in Figure 3a), there was some slight improvement for elderly males (aged 50 to 69), but the extent was much smaller than for females. For the middle-aged groups (25 to 49), the survival curve actually worsened, highlighting the need for prevention and early intervention in these age groups among Indigenous males.

## Discussion

This study quantifies the extent to which the Indigenous health gap is explained by common risk factors. The results indicate that socioeconomic and behavioural risk factors explain a large proportion of the gap in LE between Indigenous and non-Indigenous Australians. More specifically, our findings reveal that 60 to 70% of the Indigenous LE gap is attributable to six selected health risks in combination and that socioeconomic disadvantage alone is responsible for between one-third and one-half of this gap. This result is compatible with the concentration index analyses using morbidity and mortality rates [21].

Decomposition of the LE gap by health risks yields insightful information regarding the relative importance of risk factors for health inequality. This method enables more effective and efficient use of LE and mortality information. To the best of our knowledge, the LE decomposition is a novel approach to assessing risk factors and comparing the impacts of multiple competing risk factors on LE. This method allows the potential benefits of health interventions to be evaluated in terms of LE improvements.

These results are broadly comparable to those from international studies, even though they are based on different methodologies [7,28]. For example, in America the difference in LE at birth between different socioeconomic groups increased from 2.8 years in 1980 to 1982 to 4.5 years in 1998 to 2000 [29]. Socioeconomic disadvantage, smoking, high blood pressure, high blood glucose, and obesity were found to decrease LE at birth by 5 years in men and 4 years in women [7,30]. The educated live 9 to 13 years longer than the uneducated [31]. Smoking cessation will gain extra 4 to 5 years in life span [32,33]. A severe level of obesity could reduce the LE by 8 to 13 years [34]. In terms of accuracy of the results, there is little difference between this method and the Arriaga method. This approach is more effective and analytical because it can analyse the LE difference by not only proportion of cause-specific deaths, but also the mortality improvements, years of life lost, and their age-related heterogeneity [15]. Assessing risk factors using multivariate model is potentially useful for correcting upward bias introduced by the univariate models. For example, adding the single contributions of the six selected risks for males in 2001 to 2005 yields a total of 79%, whereas the combined risk is 61% (Table 3) and the bias corrected is 18%.

From 1986 to 1990 through 2001 to 2005, female Indigenous LE rose solidly by 5 years, resulting in a reduction of nearly 4 years in the LE gap. Comparatively, male Indigenous LE improved by 3 years, but the gap widened. The difference between males and females is consistent with an epidemiological transition [35]. Chronic and degenerative diseases emerge as the main causes of the gap [5]. Epidemiologic transition redistributes risks of dying from the young to the old, leading to more health care and higher cost. A mixed transition model best reflects the mortality pattern in the NT. The non-Indigenous population (70% of the NT total) is in the midst of the third demographic transition, whereas the Indigenous population is undergoing a transition from the second stage, characterized by high fertility and fast population growth. The ideal changes in survivorship, as demonstrated in Figure 3, are that the shape of the survival curve increasingly approaches a rectangular. To improve the rectangularisation for the Indigenous population, especially for Indigenous males, we need to prevent premature deaths at young and middle ages and reduce the level of age dispersion in mortality.

There are a number of limitations to the study. This study focused on mortality and LE. It did not include morbidity and quality-of-life measures. The socioeconomic measure was based on areas and assumed that the average relative risk applicable to the population by area was also applicable to individuals. The IRSAD scores are estimated by averaging the socioeconomic status of a region and the scores in the wealthiest areas of the NT are offset by the significant population of Indigenous residents from low socioeconomic areas who relocate to seek high-level health care. It was likely that IRSAD understated the true socioeconomic disadvantage for Indigenous people [36]. Due to limitations of the available data, the joint risk prevalence and lead-lag effect of preventative intervention were not examined. In addition, the study did not account for potential residual interdependencies between risks, such as the impact of alcohol abuse and assault on socioeconomic disadvantage for family members, or the possibility that relative risk varies over time. Instead, the most recent estimates of relative risk from the literature were assumed to reflect the magnitude of a particular risk across the study period independent of all other risks. The unexplained gap was about one-third, which suggests that other risk factors may be also important for explaining the gap, such as a lack of access to health care (due to residence in a remote location or barriers to primary care services, for example), and a lack of healthy food and new technology. More research is needed to further explore the effectiveness of interventions designed to reduce poverty in the Indigenous communities.

Indigenous socioeconomic disadvantage is the single most important determinant of the Indigenous LE gap. Socioeconomic status structures much of our everyday life and has a profound effect on our health. Low participation in workforce, high unemployment, low living standards, overcrowding, and lack of healthy food all have an impact on longevity. Efforts to close the Indigenous health gap will be more effective and enduring if they address the socioeconomic circumstances of Indigenous people. Conversely, the failure to address poverty in Indigenous communities is likely to undermine whatever gains might otherwise occur through traditional prevention activities, such as smoking cessation, and alcohol and obesity control campaigns.

Reducing poverty should be placed squarely at the centre of the strategy to close the Indigenous health gap. A striking fact emerging from this study is the extent to which socioeconomic disadvantage contributed to the gap relative to other behavioural risks. Attempts to modify risk behaviours without altering socioeconomic disadvantage will inevitably have limited success because the risk behaviours are often embedded within the socioeconomic disadvantage that reinforces those risk behaviours. Recognition of the relationship between socioeconomic factors and health outcomes supports efforts to eliminate poverty and improve Indigenous longevity and quality of life.

## Conclusions

Our findings highlight the striking proportional contributions to the Indigenous LE gap in Australia by socioeconomic disadvantage, smoking, and obesity. Improving socioeconomic status, smoking cessation, and overweight reduction are essential to close the Indigenous LE gap and enhance health gains for the whole population.

## Competing interests

The authors declare that they have no competing interests.

## Authors’ contributions

All authors contributed to the design of the study. YZ and JW contributed to data collection and statistical analysis. SB and SG offered technical support, and led the drafting and revision of the paper. All authors read and approved the final manuscript.
